# Real‐World Effectiveness of Nirsevimab in Preventing RSV Hospitalizations: Evidence of Protection in Southern Italian Infants, 2024–2025

**DOI:** 10.1002/jmv.70662

**Published:** 2025-10-30

**Authors:** Francesca Fortunato, Rosa Prato, Angelo Acquafredda, Angelo Campanozzi, Valeria Delli Carri, Rosa Francavilla, Giuseppina Iannelli, Pio Liberatore, Gianfranco Maffei, Matteo Mariano, Loris Micelli, Marilena Nesta, Maria Rosa Pastore, Annamaria Calvo, Matteo Rinaldi, Pier Luigi Lopalco, Domenico Martinelli

**Affiliations:** ^1^ Department of Medical and Surgical Sciences University of Foggia Foggia Italy; ^2^ Hygiene Unit, Policlinico Foggia Hospital Foggia Italy; ^3^ Pediatric Unit, Presidio Ospedaliero “Giuseppe Tatarella”, Foggia Local Health Unit Foggia Italy; ^4^ Pediatric Unit, Policlinico Foggia Hospital Foggia Italy; ^5^ Microbiology and Virology Unit, Policlinico Foggia Hospital Foggia Italy; ^6^ Pediatric Unit, Ospedale “Teresa Masselli Mascia”, Foggia Local Health Unit Foggia Italy; ^7^ Neonatology and Intensive Care Unit, Policlinico Foggia Hospital Foggia Italy; ^8^ Prevention Department Foggia Local Health Unit Foggia Italy; ^9^ Pediatric Unit, Fondazione IRCCS “Casa Sollievo della Sofferenza”, San Giovanni Rotondo Foggia Italy; ^10^ Microbiology and virology Unit, Fondazione IRCCS “Casa Sollievo della Sofferenza”, San Giovanni Rotondo Foggia Italy; ^11^ Department of Experimental Medicine University of Salento Lecce Italy

**Keywords:** Nirsevimab, real‐world effectiveness, RSV, RSV hospitalizations, South Italy

## Abstract

Respiratory syncytial virus (RSV) is a leading cause of hospitalization in infants, resulting in millions of cases and hundreds of thousands of deaths worldwide every year. Nirsevimab, a long‐acting monoclonal antibody approved in 2023, has 75%–90% protection against RSV‐related admissions. This study evaluated the effectiveness of nirsevimab in preventing RSV‐hospitalization among infants in southern Italy during 2024–2025 season. Active surveillance was conducted in all hospitals in the Foggia district. Infants born between January 2024 and March 2025 who were hospitalized with lower respiratory tract infections from October 2024 to April 2025 were tested for RSV. Information on immunoprophylaxis was provided by the Regional Immunization Information System. The effectiveness of nirsevimab was estimated using the screening method, a test‐negative case‐control design and a cohort study approach. Of the 4820 eligible infants, 2635 (54.7%) were immunized. Of the 256 infants admitted with lower respiratory tract infections, 82 tested positives for RSV; of these, 13 had received immunoprophylaxis. The effectiveness was estimated to be 84.4% (95% CI: 71.7–91.4%) using the screening method, 72.5% (42.1%–87.0%) using the test‐negative design, and 81.6% (66.4–90.0) using the cohort approach. As in previous study, these findings showed than nirsevimab provide substantial protection against RSV‐related hospitalization.

## 1 Introduction

Respiratory syncytial virus (RSV) is the leading cause of hospitalization for bronchiolitis in infants under 1 year of age [1, 2]. From January 2023, nirsevimab, a long‐acting monoclonal antibody, has been approved to provide protection for at least 5 months, which is sufficient to cover the entire RSV season and protect immunized infants [1]. Two clinical trials showed that nirsevimab was between 75% and 77% effective in preventing medically attended lower respiratory tract infections (LRTIs) and RSV‐associated hospitalizations in infants during their first RSV season [3, 4]. These findings have been supported by real‐world studies from Spain, Luxembourg, France, and the USA, which reported effectiveness rates between 82% and 90% in preventing RSV‐related hospitalizations [5, 6, 7, 8]. In the Apulia region of Southern Italy, the immunoprophylaxis campaign was launched in October 2024 and nirsevimab was offered to all infants born during the RSV epidemic season (until April 2025) and to those born between January and September 2024 who would have experienced their first RSV season in autumn/winter 2024–2025 [9].

This study shows preliminary real‐word data on the effectiveness of nirsevimab in preventing RSV‐associated hospitalizations in infant residents in the district of Foggia during the 2024–2025 season.

## 2 Methods

In all four hospitals in the district of Foggia, a hospital‐based active surveillance system was activated for all infants born between January 1, 2024 and March 31, 2025, target of the nirsevimab campaign. This cohort was derived from the Apulian Total Population Register (TPR), which contains sociodemographic data on all residents of the Foggia district, including information on sex, infant nationality and residence municipality. Within this birth cohort, all infants admitted to any of the four hospitals with a LRTI between October 1, 2024 and April 30, 2025 were tested for RSV using multiplex RT‐PCR (Allplex Respiratory Panel Assays 4). Data from the RSV surveillance were then matched with the Hospital Discharge Registry (HDR) records using unique, pseudo‐anonymized identification numbers and date of admission as linkage keys to retrieve age at hospitalisation, relevant baseline clinical characteristics (e.g., birth weight, preterm birth, comorbidities) and disease severity (e.g., co‐infections, intensive care unit— ICU admission, length of stay). The Apulian Immunization Information System (IIS), updated to March 31, 2025, provided information on nirsevimab immunoprophylaxis.

The effectiveness of nirsevimab in preventing RSV‐associated hospitalizations was estimated using the screening method, a test‐negative case‐control design, and a cohort study approach. For the screening method, the effectiveness was calculated using the formula IE = (PPI − PCI)/(PPI × (1 − PCI)), where PPI was the proportion of the population immunized, and PCI was the proportion of immunized individuals among hospitalized cases. For the test‐negative case‐control method, effectiveness was calculated as (1 − odds ratio) × 100%. In the cohort approach, the risk of RSV hospitalisation in nirsevimab‐immunised infants was compared with that in non‐immunised infants, and the absolute risk reduction (ARR) and relative risk reduction [RRR, or effectiveness = (1 − relative risk) × 100%] were calculated. The results are presented with 95% confidence intervals.

The Kruskal–Wallis test was used to analyse differences in quantitative variables, while the *χ* ^2^ test was used to compare categorical variables (sociodemographic characteristics, baseline clinical characteristics, disease severity. Multivariate logistic regression analysis was used to adjust the odds ratio (OR), with sociodemographic and baseline clinical characteristics including in the model. The relative risk (RR) was adjusted using a Poisson regression model (Supporting Information S1: SM 1). Statistics were performed using STATA 18.0 software for Mac OS. *p* values < 0.05 were considered significant.

## 3 Results

During the 2024–2025 season, a total of 2635/4820 (54.7%) infants received the nirsevimab immunoprophylaxis in the province of Foggia. Lower immunisation coverage was observed among non‐Italians than Italians (5.1% vs. 12.9%, *p* < 0.0001), as well as among infants residing outside Foggia town compared to those living in the city itself (19.7% vs. 22.5%, *p* = 0.0169 ‐ Table 1).

Between October 1, 2024 and April 30, 2025, a total of 256 infants were admitted with LRTI, of whom 82 had laboratory‐confirmed RSV (*n* = 50 males; 70%, median age at hospitalization: 4 months, see Supporting Information S1: SM 2). The RSV‐negative cases had a lower birth weight (*p* = 0.0108), whereas the RSV‐positive cases had a longer hospital stay (*p *= 0.0021) and a higher prevalence of coinfections (*p* = 0.0029). No further differences were found between cases and controls (Table 2). Of the 82 RSV‐positive patients, 14 were infected with RSV type A, 29 with type B and the other cases were of unspecified types; 26 (31.7%) had viral co‐infections. Thirteen RSV‐positive LRTI cases (15.9%) had been immunized with nirsevimab, and 2 with palivizumab. The median time interval between immunoprophylaxis and RSV‐positive test result was 38 days (one case tested positive for RSV within 7 days of the nirsevimab infection). Among hospitalized LRTIs who tested negative for RSV, the nirsevimab immunization coverage was 35.6%. The median interval between the immunoprophylaxis and testing negative for RSV was 54 days (one control tested negative for RSV within 7 days of the nirsevimab infection, Supporting Information S1: SM 3). No significant difference was found in the time elapsed between immunoprophylaxis and testing between cases and controls (*p* = 0.3519).

**Figure 1:**
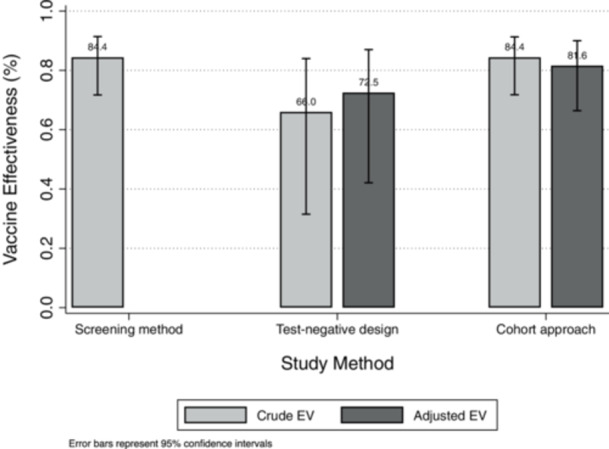
The crude and adjusted effectiveness of nirsevimab in preventing RSV‐associated hospitalisations, by study design. Foggia District, Italy, Jan1, 2024 and Apr 31, 2025.

The screening method showed that nirsevimab was 84.4% effective (95% CI: 71.7%–91.4%) in preventing RSV‐related LRTI hospitalisations. The crude effectiveness, calculated using the test‐negative design, was 66.0% (95% CI: 31.5%–84.0%), and the adjusted effectiveness was 72.5% (95% CI: 42.1%–87.0%), Supporting Information S1: SM 4). Using the cohort approach, the hospitalization risk was estimated at 0.49% (13/2635) in the immunized group versus 3.16% (69/2185) in the non‐immunized group, resulting in an ARR of 2.67% points (95% CI: 1.88%–3.45%). Nirsevimab demonstrated a crude RRR of 84.4% (95% CI: 71.8%–91.3%) and an adjusted RRR of 81.6% (95% CI: 66.4%–90.0%) (Supporting Information S1: SM 5; Figure 1).

## 4 Discussion

This analysis provides valuable insights into the effectiveness of nirsevimab in preventing RSV‐related hospitalisations in infants in the Foggia district during the 2024–2025 season, based on real‐world data. Although more than half of the infants in the target cohort received the new monoclonal antibody, this still represents suboptimal overall coverage, which is lower than that reported in other Italian regions or abroad during the first season of nirsevimab use [10, 11]. Even lower uptake was observed among infants with a foreign background and living outside Foggia town. As reported in other studies [12, 13], this suggests the persistence of socioeconomic or cultural barriers to accepting the preventive intervention and/or accessing healthcare services.

Our findings showed that nirsevimab was effective in preventing RSV‐related hospitalisations, providing immediate and lasting protection after injection. The results appeared consistent across different methods used and aligned with those reported in clinical trials and previous real‐world studies [3, 4, 5, 6, 7, 8, 14, 15, 16]. Notably, in a similar study conducted in Spain, the effectiveness of nirsevimab ranged from 69.3% to 97.0%, depending on the geographical area, using the screening method, and 70.2% (95% CI: 38.3–88.5) using the test‐negative approach [11]. A recent meta‐analysis including 32 cohort and case‐control studies from five countries (France, Italy, Luxembourg, Spain, and the USA) reported an odds ratio for the incidence of LRTI of 0.25 (95% CI 0.19–0.33) in infants aged 0–12 months [17].

This study was strengthened by comprehensive active surveillance in all four hospitals in the district of Foggia. This approach enabled the identification of a relatively small but adequate cohort of hospitalized infants, including those who had received nirsevimab, allowing robust estimates of its effectiveness using the screening method, the test‐negative case‐control design and the cohort study approach. The screening method provides a simple and rapid estimate based on aggregated population‐level data, and it is not possible to adjust for individual‐level confounders [18]. On the other hand, the test‐negative design estimates the direct effect of the immunisation intervention, allowing for control of various possible confounding factors that might influence the risk of RSV infection and the probability of being immunised [19]. In this case, however, the differences in estimates were not significant. Cohort studies represent the gold standard for measuring direct effectiveness due to their ability to control for person‐level characteristics, including age, comorbidities and behavioural factors [20]. In this study's cohort approach, the effectiveness was corrected only for the available factors from the sources used, which did not significantly affect the estimation.

This study has several methodological limitations that warrant consideration. Integrating data from disparate sources prevented the study cohort from being fully clinically characterised, particularly with regard to birth weight and gestational age parameters. The scope of the effectiveness evaluation was limited to RSV‐related hospital admissions [21], meaning the impact of immunoprophylaxis on outpatient presentations or emergency department utilisation was not assessed. The study sample size was insufficient for subgroup analyses examining effectiveness according to disease severity stratification or occurrence of serious adverse events, though such investigations were beyond the predetermined research objectives. Furthermore, the geographically restricted, single‐district methodology constrains extrapolation to populations with different demographic profiles, healthcare infrastructures, or RSV transmission dynamics.

**Table 1:** Sociodemographic characteristics of immunized (nirsevimab) versus not immunized infants. Foggia District, Italy, Jan 1, 2024 and Apr 31, 2025.

	Immunized (nirsevimab) infants (*n* = 2635)	Not immunized infants (*n* = 2185)	*p*‐value
Sex^a^			
Female	1248 (47.4%)	1078 (49.3%)	
Male	1387 (52.6%)	1107 (50.7%)	0.1722
Infant nationality^a^			
Italian	2502 (94.9%)	1904 (87.1%)	
Non‐Italian	133 (5.1%)	281 (12.9%)	**0.0000**
Residence municipality^a^			
Foggia town	593 (22.5%)	430 (19.7%)	**0.0169**
Outside Foggia town	2042 (77.5%)	1755 (80.3%)	

*Note:* Bold values indicate statistically significant at *p* < 0.05.

a Variables included in the Poisson regression model.

**Table 2:** Sociodemographic, baseline clinical characteristics, and disease severity, between RSV‐positive and RSV‐negative LRTI cases. Foggia District, Italy, Jan 1, 2024 and Apr 31, 2025.

	RSV positive (*n* = 82)	RSV negative (*n* = 174)	*p*‐value
Sex^a^			
Female	32 (39.0%)	71 (40.8%)	0.7864
Male	50 (61.0%)	103 (59.2%)	
Infant nationality^a^			
Italian	72 (87.8%)	147 (84.5%)	0.4806
Non‐Italian	10 (12.2%)	27 (15.5%)	
Residence municipality^a^			
Foggia town	18 (21.9%)	27 (15.5%)	0.2070
Outside Foggia town	64 (78.1%)	147 (84.5%)	
Birth weight^b^ (g, median [IQR])	3335 [3040–3697.5]	3190 [2755–3490]	** 0.0108 **
Birth weight^a^			
Low or high birth weight (< 2500 g or ≥ 4500 g)	4 (4.9%)	22 (12.6%)	**0.0395**
Normal birth weight (≥ 2500 g or < 4500)	74 (90.2%)	134 (77.1%)	
Preterm birth^a^,^c^			
Yes	8 (9.8%)	26 (14.9%)	0.1782
No	71 (86.6%)	130 (74.7%)	
Comorbidities^a^			
Yes	1 (1.2%)	4 (2.3%)	0.5604
No	81 (98.8%)	170 (97.7%)	
Age at hospitalization (months, median [IQR])	4 [2‐6.25]	4 [2‐8]	0.2993
Age at hospitalization^a^			
Below the median age (< 4 months)	35 (42.7%)	74 (42.5%)	0.9814
Above the median age (≥ 4 months)	47 (57.3%)	100 (57.5%)	
Time from the immunoprophylaxis to RSV test, (months, median [IQR])	38 [23–91]	54 [32.25–95]	0.3519
Length of stay (days, median [IQR])	6 [4–8]	5 [3–7]	** 0.0021 **
Co‐infections			
Yes	26 (31.7%)	27 (15.5%)	** 0.0029 **
No	56 (68.3%)	147 (84.5%)	
ICU admission			
Yes	7 (8.5%)	11 (6.3%)	0.5178
No	75 (91.5%)	163 (93.7%)	

*Note:* Bold values indicate statistically significant at *p* < 0.05.

a Variables included in the logistic regression model.

^b^ Data available for 234 infants.

^c^ Data available for 235 patients.

Despite the limitations of district‐specific preliminary data in representing broader epidemiological contexts, the concordance observed with previously published international effectiveness studies suggests that comparable protective efficacy could be achieved in other Italian regions and similar healthcare delivery systems. Continued surveillance with comprehensive data collection will be essential to validate these preliminary findings and to refine targeted public health interventions for RSV prevention.

## Declaration of Generative AI

In the preparation of this manuscript the Authors used DeepL Write and Claude 3.7 Sonet (Professional Plan) to improve readability and language of the work. After using these tools, the Authors reviewed and edited the content as necessary. The Authors take full responsibility for the content of the publication.

## Author Contributions

Conceptualization: Francesca Fortunato, Domenico Martinelli. Investigation and Data curation: Francesca Fortunato, Angelo Acquafredda, Angelo Campanozzi, Valeria Delli Carri, Rosa Francavilla, Giuseppina Iannelli, Pio Liberatore, Gianfranco Maffei, Matteo Mariano, Loris Micelli, Marilena Nesta, Maria Rosa Pastore, Annamaria Calvo, Matteo Rinaldi. Methodology and formal analysis: Francesca Fortunato, Domenico Martinelli. Supervision: Domenico Martinelli, Angelo Campanozzi, Pier Luigi Lopalco, Rosa Prato. Visualization and Validation: Francesca Fortunato, Domenico Martinelli, Pier Luigi Lopalco, Rosa Prato, Angelo Acquafredda, Angelo Campanozzi, Valeria Delli Carri, Rosa Francavilla, Giuseppina Iannelli, Pio Liberatore, Gianfranco Maffei, Matteo Mariano, Loris Micelli, Marilena Nesta, Maria Rosa Pastore, Annamaria Calvo, Matteo Rinaldi. Writing – original draft, review and editing: Francesca Fortunato, Angelo Campanozzi, Domenico Martinelli, Pier Luigi Lopalco, Rosa Prato.

## Ethics Statement

The study was conducted in accordance with the principles of the Declaration of Helsinki (1975, revised 2008). The study protocol was approved by the Local Ethics Committee “Interprovinciale Area‐I‐AOU‐Foggia‐ASL‐FG‐ASL‐BAT” (ref. 51/CE/2025).

## Conflicts of Interest

Francesca Fortunato received travel support from MSD, Sanofi, and GSK. Domenico Martinelli received personal fees as speaker from Sanofi, personal fees as advisory board member from Sanofi and Moand travel support from MSD, Sanofi, and GSK. Pier Luigi Lopalco and Rosa Prato received research grants, travel support and personal fees as advisory board member and/or speaker from MSD, Sanofi, Pfizer, GSK, Seqirus, Moderna, and Novavax. The other authors declare no conflicts of interest.

## Supporting information

EV Nirvsevibam Foggia ‐ SM ‐ 08‐09‐2025.

## Data Availability

The data that support the findings of this study are available from the corresponding author upon reasonable request.
