# Supplementary material for: Real‐World Effectiveness of Nirsevimab in Preventing RSV Hospitalizations: Evidence of Protection in Southern Italian Infants, 2024–2025
Source: J Med Virol. 2025 Oct 30;97(11):e70662. doi: 10.1002/jmv.70662 (PMC12574617; doi:10.1002/jmv.70662)
Supplement: Supplementary file 1 — EV Nirvsevibam Foggia ‐ SM ‐ 08‐09‐2025. [file JMV-97-e70662-s001.docx]

# Real-world effectiveness of nirsevimab in preventing RSV hospitalizations: evidence of protection in Southern Italian infants, 2024-2025

# Supplementary materials

## Supplementary materials 1 - Variables included for statistical, univariate and multivariate analysis

**Test negative design:** Sex (Male vs Female)*, Infant nationality (Italian vs. Non-Italian)*, Residence municipality (Foggia town vs. Outside Foggia town)*, Birth weight (g, median [IQR]; Low or high birth weight - <2500 or ≥4500 g vs. Normal birth weight - ≥2500 or <4500 g)*; Preterm birth (Yes vs. No)*; Comorbidities (Yes vs. No)*; Age at hospitalization (months, median [IQR]; Below the median age ‘<4 months’ vs. Above or equal the median age ‘≥4 months’)*; Length of stay (days, median [IQR]); Co-infections (Yes vs. No); ICU admission (Yes vs. No)

*Variables included in logistic model.

**Cohort approach (Poisson model):** Sex (Male vs. Female), Infant nationality (Italian vs. Non-Italian), Residence municipality (Foggia town vs. Outside Foggia town)

## Supplementary materials 2

## LRTI distribution by age, nirsevimab immunization status and laboratory result for RSV. Test negative design, Foggia District, Italy, 1-Jan-2024 and 30-Apr-2025.

|  |
| --- |
|  |

## Supplementary materials 3

## LRTI distribution by interval between the immunoprophylaxis and testing. Test negative design, Foggia District, Italy, 1-Jan-2024 and 30-Apr-2025.

## Supplementary materials 4 – Logistic model to adjust effectiveness in test negative design

| Logistic regression | Number of obs: 234 |
| --- | --- |
|  | LR chi2(7): 19.51 |
|  | Prob > chi2: 0.0124 |
| Log likelihood =-139.18869 | Pseudo R2: 0.0655 |

| **Variables** | **Odds ratio** | **Std. err.** | **z** | **P>z** | **[95% conf. interval]** | |
| --- | --- | --- | --- | --- | --- | --- |
| Immunization status  *Immunized with nirsevimab vs Not immunized* | 0.2747947 | 0.1044744 | -3.40 | **0.001** | 0.1304335 | 0.5789316 |
| Sex  *Male vs. Female* | 0.9737184 | 0.2918958 | -0.09 | 0.929 | 0.5410844 | 1.752273 |
| Infant nationality  *Italian vs. non-Italian* | 1.506084 | 0.6415143 | 0.96 | 0.336 | 0.6535538 | 3.470702 |
| Residence municipality  *Foggia town vs Outside Foggia town* | 1.4039 | 0.5250208 | 0.91 | 0.364 | 0.674543 | 2.921882 |
| Birth weight  *Low or high birth weight (<2500 or ≥4500 g) vs. Normal birth weight (≥2500 g or <4500)* | 3.392531 | 2.886062 | 1.44 | 0.151 | 0.640321 | 1.797421 |
| Preterm birth  *Yes vs. No* | 1.141633 | 0.7841523 | 0.19 | 0.847 | 0.2970726 | 4.387231 |
| Comorbidities  *Yes vs. No* | 0.7718397 | 0.9360577 | -0.21 | 0.831 | 0.071651 | 8.314417 |
| Age at hospitalization  *Yes vs. No* | 0.817145 | 0.2542644 | -0.65 | 0.516 | 0.4440559 | 1.503698 |
| _cons | 0.165977 | 0.1664927 | -1.79 | 0.073 | 0.0232382 | 1.18548 |

## Supplementary materials 5 – Poisson model to adjust effectiveness in test cohort approach

| Iteration 0: Log pseudolikelihood: | -349.1949 |
| --- | --- |
| Iteration 1: Log pseudolikelihood | -349.19361 |
| Iteration 2: Log pseudolikelihood | -349.19361 |
|  |  |
| Poisson regression | Number of obs: 4,809 |
|  | Wald chi2(4): 31.05 |
|  | Prob > chi2: 0.0000 |
| Log pseudolikelihood = -349.19361 | Pseudo R2: 0.0570 |

| **Variables** | **IRR** | **Robust std. err.** | **z** | **P>z** | **[95% conf. interval]** | |
| --- | --- | --- | --- | --- | --- | --- |
| Immunization status  *Immunized (nirsevimab) vs Not immunized* | 0.1835923 | 0.0567078 | -5.49 | **0.000** | 0.1002158 | 0.3363355 |
| Sex  *Male vs, Female* | 1.395817 | 0.3352606 | 1.39 | 0.165 | 0.871724 | 2.235004 |
| Infant nationality  *Italian vs, non-Italian* | 1.047731 | 0.3941216 | 0.12 | 0.901 | 0.5012518 | 2.189997 |
| Residence municipality  *Foggia town vs. Outside Foggia town* | 0.8972453 | 0.2742923 | -0.35 | 0.723 | 0.4928273 | 1.633532 |
| _cons | 0.0217824 | 0.0083085 | -10.03 | 0.000 | 0.0103141 | 0.0460025 |
